# E3 Ubiquitin Ligase Synoviolin Is Involved in Liver Fibrogenesis

**DOI:** 10.1371/journal.pone.0013590

**Published:** 2010-10-25

**Authors:** Daisuke Hasegawa, Ryoji Fujii, Naoko Yagishita, Nobuyuki Matsumoto, Satoko Aratani, Toshihiko Izumi, Kazuko Azakami, Minako Nakazawa, Hidetoshi Fujita, Tomoo Sato, Natsumi Araya, Junki Koike, Mamoru Tadokoro, Noboru Suzuki, Kazuhiro Nagata, Haruki Senoo, Scott L. Friedman, Kusuki Nishioka, Yoshihisa Yamano, Fumio Itoh, Toshihiro Nakajima

**Affiliations:** 1 Institute of Medical Science, St. Marianna University School of Medicine, Kawasaki, Japan; 2 Division of Gastroenterology and Hepatology, St. Marianna University School of Medicine, Kawasaki, Japan; 3 Department of Pathology, St. Marianna University School of Medicine, Kawasaki, Japan; 4 Departments of Immunology and Medicine, St. Marianna University School of Medicine, Kawasaki, Japan; 5 Department of Molecular and Cellular Biology, Institute for Frontier Medical Sciences, Kyoto University, Kyoto, Japan; 6 Department of Cell Biology and Histology, Akita University School of Medicine, Hondo, Japan; 7 Division of Liver Diseases, Mount Sinai School of Medicine, New York, New York, United States of America; 8 Institute of Medical Science, Tokyo Medical University, Tokyo, Japan; 9 Choju Medical Institute Fukushimura Hospital, Toyohasi, Japan; 10 Misato Marine Hospital, Kochi, Japan; City of Hope National Medical Center, United States of America

## Abstract

**Background and Aim:**

Chronic hepatic damage leads to liver fibrosis, which is characterized by the accumulation of collagen-rich extracellular matrix. However, the mechanism by which E3 ubiquitin ligase is involved in collagen synthesis in liver fibrosis is incompletely understood. This study aimed to explore the involvement of the E3 ubiquitin ligase synoviolin (Syno) in liver fibrosis.

**Methods:**

The expression and localization of synoviolin in the liver were analyzed in CCl_4_-induced hepatic injury models and human cirrhosis tissues. The degree of liver fibrosis and the number of activated hepatic stellate cells (HSCs) was compared between wild type (wt) and Syno^+/−^ mice in the chronic hepatic injury model. We compared the ratio of apoptosis in activated HSCs between wt and Syno^+/−^ mice. We also analyzed the effect of synoviolin on collagen synthesis in the cell line from HSCs (LX-2) using siRNA-synoviolin and a mutant synoviolin in which E3 ligase activity was abolished. Furthermore, we compared collagen synthesis between wt and Syno^−/−^ mice embryonic fibroblasts (MEF) using quantitative RT-PCR, western blotting, and collagen assay; then, we immunohistochemically analyzed the localization of collagen in Syno^−/−^ MEF cells.

**Results:**

In the hepatic injury model as well as in cirrhosis, synoviolin was upregulated in the activated HSCs, while Syno^+/−^ mice developed significantly less liver fibrosis than in wt mice. The number of activated HSCs was decreased in Syno^+/−^ mice, and some of these cells showed apoptosis. Furthermore, collagen expression in LX-2 cells was upregulated by synoviolin overexpression, while synoviolin knockdown led to reduced collagen expression. Moreover, in Syno^−/−^ MEF cells, the amounts of intracellular and secreted mature collagen were significantly decreased, and procollagen was abnormally accumulated in the endoplasmic reticulum.

**Conclusion:**

Our findings demonstrate the importance of the E3 ubiquitin ligase synoviolin in liver fibrosis.

## Introduction

All forms of chronic hepatic damage ultimately result in liver cirrhosis or fibrosis, which is among the important causes of morbidity and mortality worldwide. Cirrhosis is essentially late-stage fibrosis triggered by chronic liver damage from various causes, including hepatitis virus infection, alcohol abuse, or nonalcoholic steatohepatitis [1]. Liver fibrosis can progress to widespread distortion of the normal hepatic architecture as a result of continuous liver damage and regeneration. Thus, controlling liver fibrosis is important for preventing the development of liver cirrhosis. However, currently, there are no approved anti-fibrotic therapies for liver cirrhosis, underscoring the importance of clarifying the underlying pathogenetic mechanisms.

The principal resident liver cells that drive liver fibrosis are hepatic stellate cells (HSCs), i.e., perisinusoidal cells whose primary role in the normal liver is the uptake and storage of vitamin A (retinoids) [1], [2]. In the adult liver, quiescent HSCs are located in the space of Disse between hepatocytes and sinusoidal endothelial cells. They play a pivotal role in liver physiology; following liver damage, HSCs become “activated,” i.e., they differentiate into myofibroblasts, proliferate, and produce an extracellular matrix (ECM) network mainly comprising collagen, which is the hallmark of a fibrotic scar [3]. Following acute damage, activated HSCs probably promote hepatocyte proliferation and organ repair [4], [5]; however, following chronic damage, the excessive ECM produced by these cells disrupts the hepatic cytoarchitecture, eventually leading to fibrosis and cirrhosis [1]. Therefore, pathways regulating collagen synthesis by activated HSCs in liver fibrosis represent a critical area for further investigation.

Collagen I is a major component of the extracellular matrix essential for supporting and organizing most tissues. The collagen I molecule is a trimer of two pro-α1(I) chains and one pro-α2(I) chain; the triple helix formation of the collagen occurs in the endoplasmic reticulum (ER) [6]. Further, collagen I regulates several posttranslational modifications [6]. During procollagen biosynthesis in the ER, several molecular chaperones assist in the correct folding of collagen [7]. The procollagen molecules that are fully modified and folded are then transported to the Golgi apparatus. In the Golgi cisternae, the procollagen molecules are stacked laterally, form aggregates and are further modified for the sorting to their final destinations. Finally, the procollagen aggregates are secreted into the extracellular space, where the N- and C-propeptides are enzymatically cleaved off, thereby generating mature collagen molecules [8]. Thus, the collagen triple helix must be correctly folded to allow its secretion from the cell. Collagen I chains containing mutations that affect initial chain association, such as those in the pro-α1(I), are removed by retrotranslocation of monomeric unfolded mutant collagen chains into the cytosol, followed by ER-associated degradation (ERAD)—an ATP-dependent ubiquitin-proteasome process that reduces the burden of excess unfolded proteins on the ER [9]. These proteasomal degradation systems are also involved in the collagen I synthesis of HSCs [10]. However, there is no evidence that in the ERAD system, the E3 ubiquitin ligase directly ubiquitinates unfolded collagen I chain [9].

We previously characterized a novel molecule termed synoviolin, which is strongly expressed in rheumatoid synovial fibroblasts and contributes to the pathogenesis of rheumatoid arthritis (RA) [11]–[16]. Synoviolin is an E3 ubiquitin ligase, which is a human homologue of the murine ER-resident RING-H2 ubiquitin ligase Hrd1p/Der3p [17]–[20]. The ER plays an important role in protein folding and processing. When the level of unfolded proteins in the ER exceeds the folding capacity of this organelle, defective proteins are eliminated by the ERAD system [9], in which the E3 ubiquitin ligase synoviolin is involved. E3 ubiquitin ligases catalyze ubiquitination, which can tag specific proteins for degradation [21]. Synoviolin is ubiquitously distributed among mammals, and in humans, it is expressed most strongly in the liver among all tissues [19]. We have demonstrated that synoviolin-deficient homozygous (Syno^−/−^) mice suffer death around embryonic day 13.5 (E13.5) because they have a hematopoietic abnormality provoked by hepatocellular apoptosis [22].

On the basis of these observations, we hypothesized that synoviolin may also play important roles in physiological and various pathological conditions of the liver. Here, we have explored the involvement of synoviolin in liver fibrosis using a mouse model and in human liver cirrhosis.

## Materials and Methods

### Antibodies

The antibodies used in this study were as follows: anti-synoviolin/Hrd1 monoclonal antibody as described previously [18], anti- Synoviolin/HRD1 polyclonal antibody (Santa Cruz Biotechnology, Inc.), anti-alpha smooth muscle actin (α-SMA) monoclonal antibody (Dako), anti-Flag antibody (anti-Flag M2 monoclonal antibodies; Sigma Chemical Co.), anti-KLF6/Zf9 antibody (R-173; Santa Cruz), anti-mouse collagen type I polyclonal antibody (AB765P; Millipore), anti-GM130 monoclonal antibody (BD Bioscience, Franklin Lakes, NJ), anti-protein disulfide isomerase (PDI) monoclonal antibody (Stressgen) and anti-β-actin antibody (Sigma).

### Isolation of MEFs

Mouse embryonic fibroblasts (MEFs) were isolated from E12.5 embryos of wild-type and Syno^−/−^ mice as described previously [22].

### Preparation of cellular protein and immunoblot analysis

Proteins were extracted from MEFs and LX-2 cells using a cell extraction buffer containing 0.05 M Tris-HCl, pH 8.0, 0.15 M NaCl, 5.0 mM ethylenediaminetetraacetic acid (EDTA), 1% NP-40, and protease inhibitors (1 µg/ml leupeptin and pepstatin) at 4°C. The protein extracts were resolved by 8–10% SDS-PAGE, transferred onto a nitrocellulose membrane, and incubated with primary antibodies followed by horseradish peroxidase-conjugated secondary antibodies. The antigen-antibody complexes were visualized using an ECL detection system (Promega).

### Measurement of soluble secreted collagen

Cells were seeded in low-glucose Dulbecco's Modified Eagle Medium (DMEM) containing 1% fetal bovine serum (FBS) at 24 h before collagen measurement. The total soluble collagen in the culture supernatants was measured by the Sircol collagen assay method (Biocolor) according to the manufacturer's instructions. We measured the amount of soluble collagen by subtracting the amount of soluble collagen within 1% FBS medium from the amount of total soluble collagen, and expressed it as the “relative secreted protein” by dividing the amount of soluble collagen by the amount of total protein in each sample. The assay was performed in triplicate, and the mean values of each sample were calculated.

### Cell culture and transient transfection

The LX-2 cell line was established and characterized as described previously [23].

LX-2 cells were cultured in low-glucose DMEM. The medium was supplemented with 1% penicillin, 1% streptomycin, and 10% FBS. The cells were seeded in low-glucose DMEM containing 0.5% FBS at 24 h before the transient transfection experiments. Transient transfections of the LX-2 cell line were performed using FuGENE 6 (Roche) according to the manufacturer's instructions. LX-2 cells (2×10^5^) were seeded into each well of a 6-well plate or 1×10^6^ cells were seeded into 100-mm dishes and grown overnight.

### RNA interference assay

siRNA with 21 nucleotides was chemically synthesized at Hokkaido System Science (Hokkaido, Japan). The sequences of the synoviolin siRNA oligoribonucleotides were as previously described [24]. Next, 50 nmol of annealed RNA duplex was transfected using Lipofectamine 2000 according to the manufacturer's recommendations. Firefly-scrambled synoviolin siRNA was used as the negative control as described previously [24].

### Primers

The primers used to amplify the coding sequences in the PCR were as follows (sense and antisense): mouse klf6: 5′-CCTGGAGGAATATTGGCAAC-3′ and 5′-AGGTCTTCCTGGCTGTCAAA-3′; human synoviolin: 5′-TTCGTCAGCCACGCCTAT-3′ and 5′-GAGCACCATCGTCATCAGG-3′; mouse synoviolin: 5′-TACCTCACTGTGCTGGCTTC-3′ and 5′-AAGGGGCAGCAGATACCAC-3′; mouse Acta2: 5′-GACACCACCCACCCAGAGT-3′ and 5′-ACATAGCTGGAGCAGCGTCT-3′; human COL1A1: 5′-CCCCTGGAAAGAATGGAGAT-3′ and 5′-AATCCTCGAGCACCCTGA-3′; mouse Col1al: 5′-CCCCTGGAAAGAATGGAGAT-3′ and 5′-AATCCTCGAGCACCCTGA-3′; human β-ACTIN: 5′-CCAACCGCGAGAAGATGA-3′ and 5′-TCCATCACGATGCCAGTG-3′; mouse β-actin: 5′-AAGGCCAACCGTGAAAAGAT-3′ and 5′-GTGGTACGACCAGAGGCATAC-3′; and 18S rRNA: 5′-GCTGCTTTAAGACCTACCGATG-3′ and 5′-GGATCAAGTTCACAGGCAACTA-3′.

### Real-time PCR

Total RNA from MEFs or LX-2 cells was isolated using Isogen (Nippon Gene, Tokyo, Japan), and cDNA was synthesized. Real-time PCR relative quantification analysis was performed using the FastStart Universal Probe Master (Rox; Roche Applied Science, Indianapolis, IN) and probes from the universal probe library set (Roche) with the ABI Prism 7500 Sequence Detection System and software, according to the manufacturer's recommendations (Applied Biosystems). The mRNA level was normalized relative to the amount of the transcript of β-actin, a housekeeping gene, or 18S rRNA.

### Plasmid construction

The constructions of pcDNA3/synoviolin wt-FLAG or C307S-FLAG and pcDNA3 plasmids have been described previously [18], [22], [24], [25]; similarly, the construction of KLF6 wt has also been described [26].

### Mouse models

Synoviolin-deficient mice were generated as described previously [18]. Wt C57/BL6 mice were obtained from the Jackson Laboratory. These mice were 6–7 weeks of age. To generate an acute hepatic injury model, wt mice were administered a single dose of 2.0 mL/kg 50% CCl_4_ in olive oil. They were then sacrificed 3–72 h after the injection, and their livers were used for further analysis as previously described [27].

To generate a liver fibrosis model, wt and Syno^+/−^ mice were injected with 0.5% phenobarbital water for pretreatment and then 10.0 mL/kg 2.5% w/v CCl_4_ or pure olive oil, twice a week at equal intervals for 3 weeks. All the treated mice were sacrificed 3 weeks after the first administration of CCl_4_. We sacrificed these chronic hepatic injury model mice 48 h after the final CCl_4_ exposure. Liver tissue sections were stained with Masson's trichrome stain. For the semiquantitative analysis of fibrosis, the blue-stained area in Masson's trichrome-stained sections was measured using the NIH Image software. Three fields were randomly selected from each of the 3 sections.

### Immunohistochemistry

Tissue blocks were cut (approximately 10 mm ×10 mm ×10 mm) and immediately fixed in 10% neutral buffered formalin for 48 h. After fixation, the tissues were immersed in 70% ethanol until processing. All tissues were processed simultaneously. The fixed tissues were dehydrated in ethanol, cleared in xylene, and embedded in paraffin blocks, which were cooled before sectioning. The mouse livers were sectioned into 4-µm-thick slices, and the sections were mounted on silane-coated slides. For antigen retrieval, the slides were heated in a microwave oven and then allowed to cool. Endogenous peroxidase was blocked with 3% H_2_O_2_ for 15 min at room temperature. Immunohistostaining was performed using antibodies against synoviolin (10 µg/mL) (Santa Cruz). Immunoreactive materials were visualized using a biotinylated anti- rabbit IgG antibody (Dako), peroxidase-labeled streptavidin, and diaminobenzidine. Pathologically confirmed human liver tissue arrays were obtained from U.S. Biomax (Rockville, MD).

### Double immunofluorescence staining for liver tissues and culture cells

Incubations with primary antibodies, i.e., rabbit antibody (anti-Synoviolin/HRD1 polyclonal antibody or anti-mouse collagen type I polyclonal antibody) and mouse monoclonal antibody (anti-α-SMA monoclonal antibody, anti-GM130 monoclonal antibody, or anti-PDI monoclonal antibody), were conducted at room temperature for 1 h. After washing, cells were incubated with biotinylated anti-rabbit immunoglobulin (IgG (1∶150; Vector Laboratories, Burlingame, CA, USA) and Alexa Fluor 594 (red) anti-mouse IgG (1∶150; Invitrogen) at room temperature for 1 h. After washing, reaction with streptavidin and Alexa Fluor 488 (green) conjugate (1∶150; Molecular Probes, Eugene, OR, USA) was conducted at room temperature for 1 h. After washing, coverslips were mounted using Vectashield medium with 4′,6-diamidino-2-phenylindole (Roche Diagnostics, Mannheim, Germany). Fluorescent images were visualized using a Zeiss LSM 510 META confocal fluorescence microscope (Carl Zeiss, Jena, Germany).

### TUNEL staining

For detection of cells undergoing apoptosis, tissue sections were subjected to terminal dUTP nick-end labeling (TUNEL), using an In Situ Apoptosis Detection kit (MK-500; Takara Shuzo Co., Tokyo, Japan), according to the manufacturer's recommendations. Paraffin-embedded tissue sections of rat mammary gland were used as a positive control [28] (Fig. S1). After TUNEL staining, sections were subsequentlyincubated with monoclonal anti-human α-SMA antibody and Alexa Fluor 594 (red) anti-mouse IgG (1∶150; Invitrogen) at room temperature for 1 h. The number of double-positive cells for TUNEL and α-SMA was counted in the stained sections at 400-fold field magnification under a microscope, using 5 randomly selected microscopic fields per section.

### Statistical analysis

Statistical analysis was performed using a commercially available software package (Prism 5.0; Graphpad Software). Data were tested by one-factor ANOVA with Tukey's post-hoc analysis or Student's *t*-test. Differences were considered statistically significant at *P*<0.05. All results were derived from at least 3 independent experiments.

### Ethical considerations

All experiments with mice were approved by the ethics committee for Animal Experiments of St. Marianna University School of Medicine. All the experimental protocols described in this study were approved by the Ethics Review Committee of St. Marianna University School of Medicine (Approval number 01008).

## Results

### Increased expression of synoviolin in activated HSCs in hepatic injury

**Figure 1:**
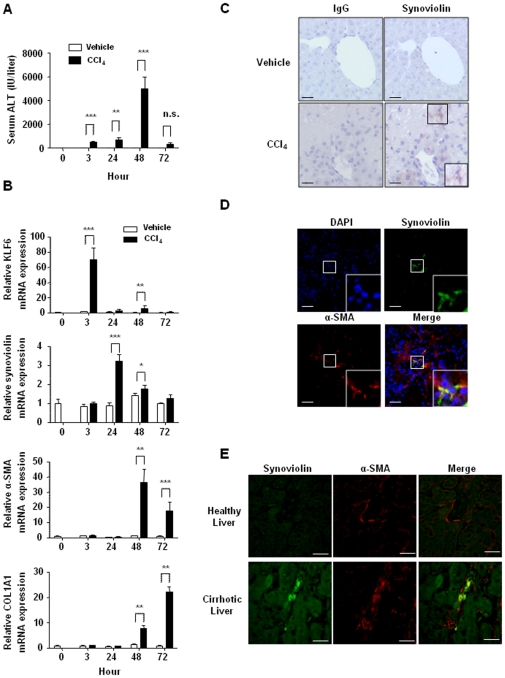
*In vivo* assessment of synoviolin expression in hepatic injury. (A) The serum levels of alanine aminotransferase (ALT) were analyzed at 0, 3, 24, 48, and 72 h after CCl_4_ administration in the acute hepatic injury model mice. Data are represented as mean ± SEM (n = 4–5 mice per group). Unpaired Student's *t*-test was used for statistical analysis. *** *P*<0.001, ** *P*<0.01. (B) Time-course expression of KLF6, synoviolin, Acta2 (α-SMA), and COL1A1 (collagen I) mRNA in livers inoculated with CCl_4_ (black bar) or vehicle (white bar) quantified using real-time RT-PCR. Data are represented as mean ± SEM (n = 4–5 mice per group). The mRNA level was normalized relative to the amount of the transcript of 18S rRNA, a housekeeping gene. Unpaired Student's *t*-test was used for statistical analysis. *** *P*<0.001, ** *P*<0.01, * *P*<0.05. (C) Immunohistochemical analysis of liver sections of wt mice at 48 h after treatment with CCl_4_ (n = 4) or vehicle (n = 5). The expression and localization of synoviolin (arrows, right panel) were analyzed using anti-synoviolin antibodies. Normal IgG antibody was used for the negative control (left panels). Scale bar  = 100 µm. (D) Double-labeled fluorescent immunohistochemical analysis of liver sections of wt mice at 48 h after treatment with CCl_4_ (n = 4). The nuclei were counterstained with DAPI (Vectashield). The expression and localization of synoviolin (green) and α-SMA (red)—a marker of activated HSCs, were analyzed using anti-synoviolin and α-SMA antibodies. Scale bar  = 200 µm. (E) Double-labeled fluorescent immunohistochemical analysis of liver sections of human healthy liver (n = 30) and cirrhotic liver (n = 40) using human tissue array sections. The expression and localization of synoviolin (green label) α-SMA (red label) were analyzed using anti-synoviolin and α-SMA antibodies. Scale bar  = 100 µm.

First, we examined whether synoviolin is expressed under pathophysiological conditions by using an acute hepatic injury model induced by CCl_4_ administration. After confirming acute liver damage by the increased serum levels of alanine aminotransferase (ALT) (Fig. 1A), we analyzed the time-course of mRNA expression for synoviolin, Krüppel-like factor 6 (KLF6)—a key transcriptional factor contributing to liver fibrosis [29], α-SMA-activated HSC marker, and the collagen I gene (*COL1A1*) using real-time quantitative reverse transcription polymerase chain reaction (RT-PCR) (Fig. 1B). As has been reported previously, rapid expression of KLF6 mRNA was observed 3 h after CCl_4_ administration, which decreased subsequently (Fig. 1B). Maximum expression of synoviolin mRNA was observed at 24 h, after which it gradually decreased (Fig. 1B). Increased expression of α-SMA mRNA was noted at 48 h (Fig. 1B), and the expression of COL1A1 mRNA increased after 72 h (Fig. 1B). This parallel expression of synoviolin and α-SMA mRNA (Fig. 1B) in liver tissues suggests that the expression of synoviolin is potentially induced along with the processes of HSCs activation in acute hepatic injury. Therefore, to determine whether the expression of synoviolin is associated with HSCs activation in hepatic injury, we initially analyzed the expression of synoviolin by immunohistochemistry in the liver 48 h after CCl_4_ administration. Synoviolin was highlyexpressed in hepatic nonparenchymal cells, among which HSCs are a prominent component (Fig. 1C). To localize the expression in the HSCs, we analyzed the dual expression of synoviolin and the activated HSC marker α-SMA in liver sections by double-labeled fluorescent immunohistochemical analysis (Fig. 1D). The sites of synoviolin expression were in part co-localized with α-SMA (Fig. 1D). Furthermore, to determine whether synoviolin is induced in the activated HSCs of human cirrhosis patients, we analyzed the co-localization of synoviolin and α-SMA in healthy and cirrhotic liver sections by double-labeled fluorescent immunohistochemical analysis using a liver human tissue array (Fig. 1E). The sites of synoviolin expression were co-localized with α-SMA in cirrhotic livers but not in healthy livers (Fig. 1E). These results suggest that synoviolin is overexpressed in activated HSCs both in the acute phases of hepatic injury mice model and in the chronic phase of human hepatic injury.

### Syno^+/−^mice are resistant to liver fibrogenesis

**Figure 2:**
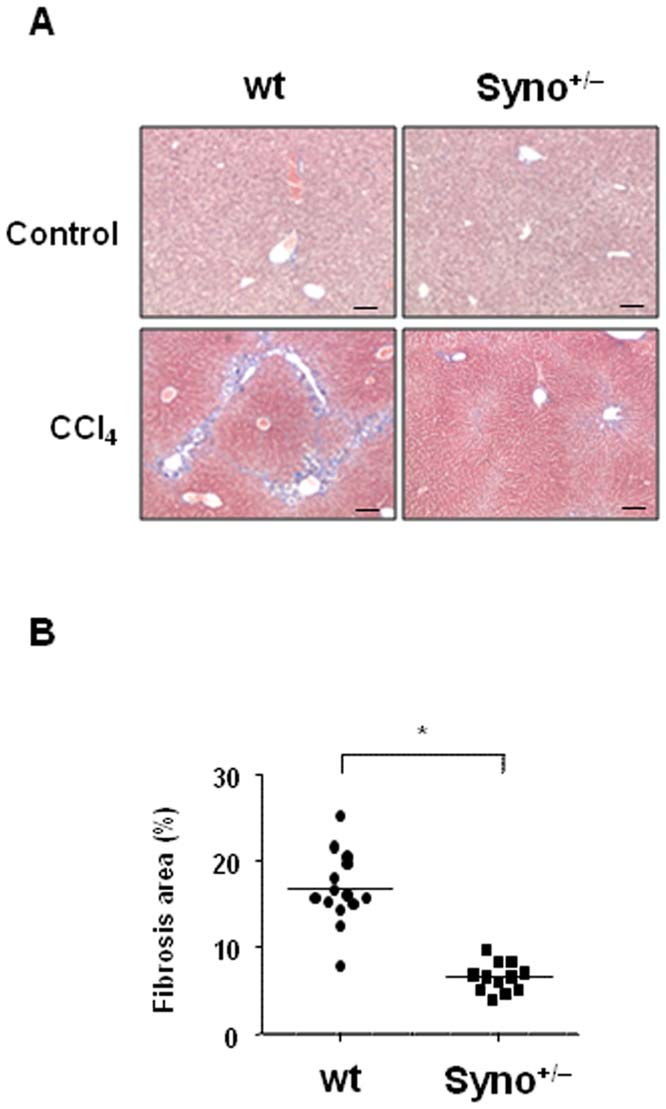
Decreased liver fibrogenesis in Syno^+/−^ mice. (A) Liver fibrosis in wt and Syno^+/−^ mice. Wild-type mice (wt: n = 14) and Syno^+/−^ mice (Syno^+/−^; n = 12) were injected with CCl_4_ twice a week for 3 weeks. Then, liver fibrosis was evaluated by Masson's trichrome staining. Wild-type mice (wt: n = 2) and Syno^+/−^ mice (Syno^+/−^; n = 2) that were treated with vehicle were used as negative controls (upper panels). Scale bar  = 200 µm. (B) Scatter diagram analysis of fibrosis areas in liver sections semiquantified using NIH Image software. Data are represented as mean ± SEM (wt: n = 14, Syno^+/−^: n = 12 of the CCl_4_-induced chronic hepatic injury model). Unpaired Student's *t*-test was used for statistical analysis. * *P*<0.001.

Because activated HSCs are considered key players in the development of liver fibrosis, we investigated whether synoviolin is essential to the development of liver fibrosis. Therefore, using a liver fibrosis model induced by chronic administration of CCl_4_, we compared the degree of liver fibrosis between wt (n = 14) and Syno^+/−^ mice (n = 12). For Syno^+/−^ mice, it is known that the expression level of synoviolin is approximately half as compared to that in wt mice [18] (Fig. 2). While liver fibrogenesis was apparent with the formation of bridging fibrosis, i.e., interlobular connective tissue fibrosis, was significant in the wt mice, fibrosis was significantly reduced in the Syno^+/−^ mice following the chronic administration of CCl_4_ (Fig. 2A). Moreover, the percentage of the fibrotic area as assessed using NIH Image software was significantly lower in the Syno^+/−^ mice than in wt mice (Fig. 2B). There was no significant difference in the serum levels of ALT, lactate dehydrogenase (LDH), total bilirubin (T-bil), total protein (TP), albumin (Alb), and the serum albumin/globulin (A/G) ratio between the wt and Syno^+/−^ mice both in natural conditions and in this liver fibrosis model (Fig. S2 and S3). These similar serum levels of ALT elevation suggest that the degree of chronic hepatic injury is almost identical between wt and Syno^+/−^ mice. However, despite the similar levels of chronic hepatic injury, the degree of liver fibrogenesis was lower in the Syno^+/−^ mice as compared with than in wt mice (Fig. 2).

### Increased number of apoptotic HSCs in liver fibrosis lesions of Syno^+/−^ mice

**Figure 3:**
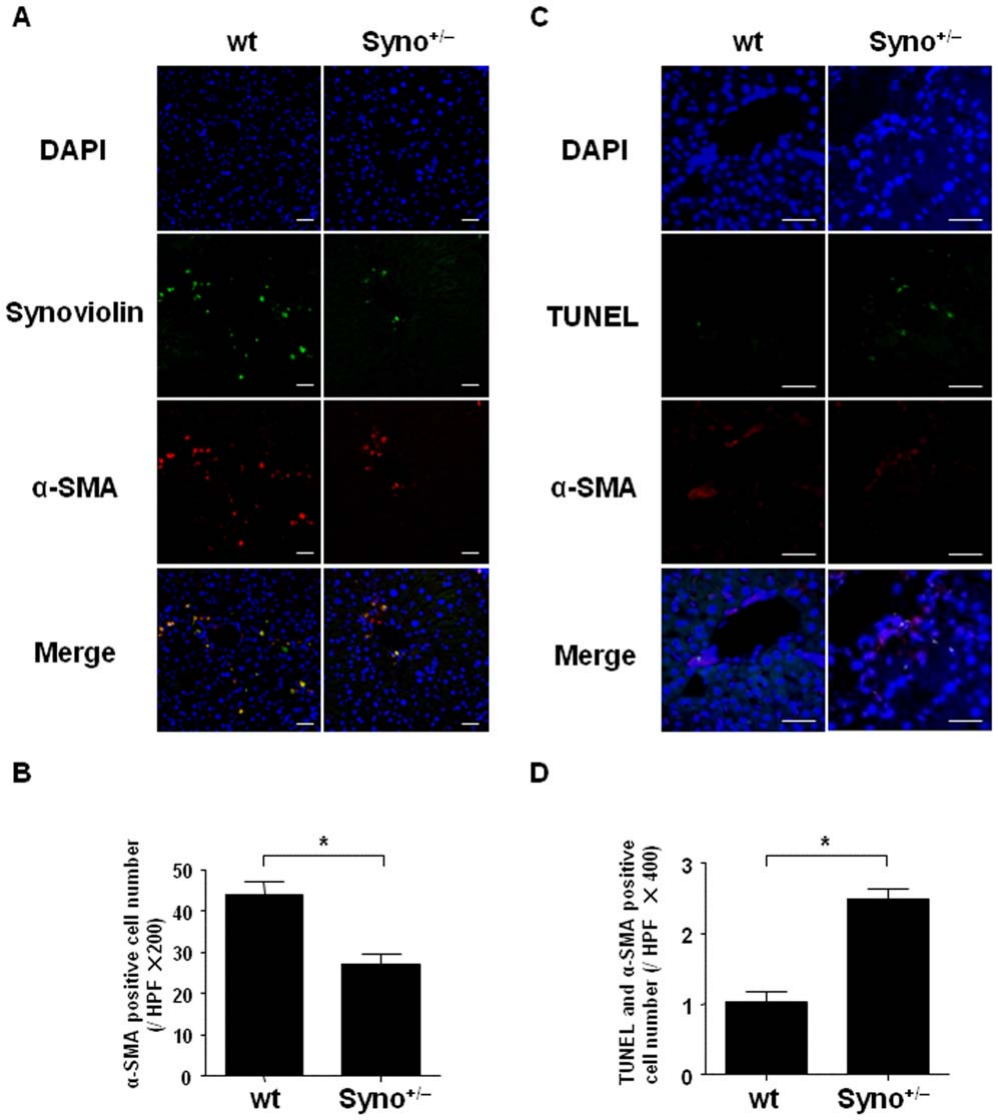
Increase in the number of apoptotic activated hepatic stellate cells in Syno^+/−^ mice. (A) Double-labeled fluorescent immunohistochemical analysis of liver sections of wt (n = 14) and Syno^+/−^ mice (n = 12) after treatment with CCl_4_ twice a week for 3 weeks. The nuclei were counterstained with DAPI (Vectashield). The expression and localization of synoviolin (green) and α-SMA (red) were analyzed using anti-synoviolin and α-SMA antibodies. Scale bar  = 100 µm. (B) The analysis of the number of α-SMA-positive cells in liver sections of wt and Syno^+/−^ mice after treatment with CCl_4_ twice a week for 3 weeks. Data are represented as mean ± SEM (wt: n = 14, Syno^+/−^: n = 12 of the CCl_4_-induced chronic hepatic injury model). Unpaired Student's *t*-test was used for statistical analysis. * *P*<0.01. (C) Double-labeled fluorescent immunohistochemical analysis of liver sections of wt and Syno^+/−^ mice after treatment with CCl_4_ twice a week for 3 weeks. The nuclei were counterstained with DAPI (Vectashield). The expression and localization of TUNEL (green) and α-SMA (red) were analyzed using an *in situ* apoptosis assay kit and α-SMA antibodies. Scale bar  = 100 µm. (D) The analysis of the number of TUNEL and α-SMA double-positive cells in liver sections of wt and Syno^+/−^ mice after treatment with CCl_4_ twice a week for 3 weeks. Data are represented as mean ± SEM (wt: n = 14, Syno^+/−^: n = 12 of the CCl_4_-induced chronic hepatic injury model). Unpaired Student's *t*-test was used for statistical analysis. * *P*<0.01.

Based on the induction of synoviolin in activated HSCs (Fig. 1) and the decreased fibrosis area in Syno^+/−^ mice compared to wt mice (Fig. 2), we hypothesized the two following possibilities: (1) synoviolin may protect activated HSCs from apoptosis, which then contributed to fibrogenesis because synoviolin is known to have an anti-apoptotic effect [12] or (2) synoviolin is directly involved in collagen production in activated HSCs. Although these possibilities are not mutually exclusive, to investigate whether synoviolin expression alters the apoptosis of activated HSCs between wild-type and synoviolin+/− mice, we initially analyzed the number of activated HSCs (α-SMA-positive cells) in liver sections between wt (n = 14) and Syno^+/−^ mice (n = 12) using a liver fibrosis model induced by the chronic administration of CCl_4_ (Fig. 3A). The partial co-localization of synoviolin and α-SMA was also demonstrated in the chronic phase (Fig. 3A), and the number of α-SMA-positive cells was decreased in the Syno^+/−^ mice as compared to that in the wt mice (Fig. 3B). Furthermore, we analyzed the dual expression of TUNEL and α-SMA in liver sections of the chronic liver fibrosis model using wt and synoviolin+/− mice by double-labeled fluorescent immunohistochemical analysis, and compared the number of TUNEL and α-SMA double-positive cells between wt (n = 14) and Syno^+/−^ mice (n = 12). As shown in Fig. 3C and 3D, the number of apoptotic HSCs was increased in Syno^+/−^ mice, suggesting that Syno^+/−^ mice are resistant to liver fibrosis following the enhanced apoptosis of HSCs.

### Synoviolin regulates secreted collagen expression in activated HSCs

**Figure 4:**
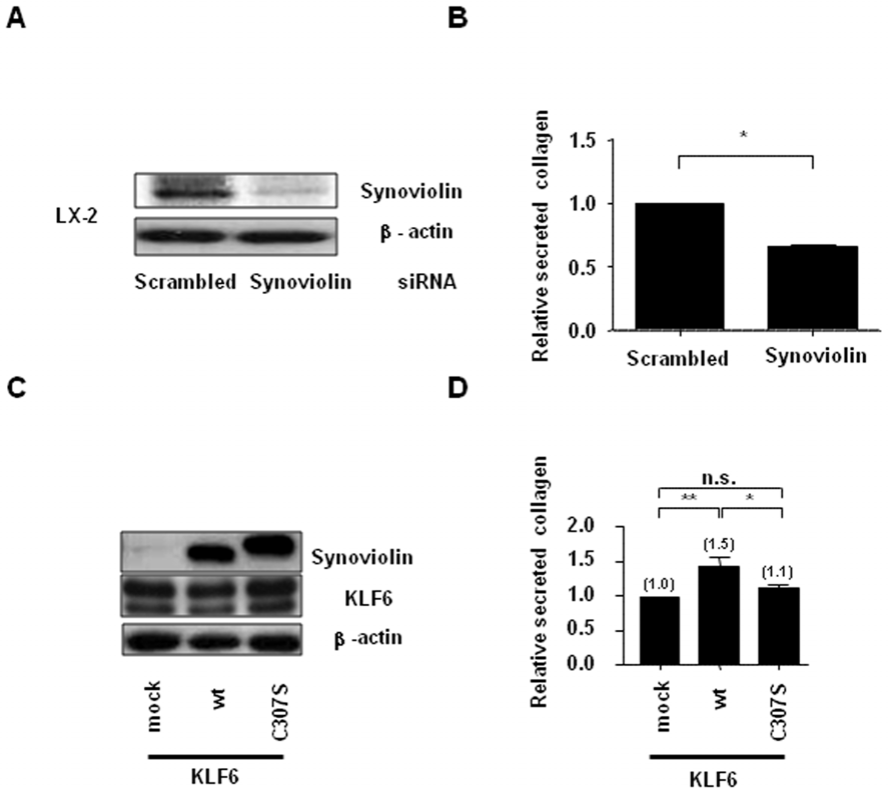
Regulation of secreted collagen expression in culture cells by synoviolin ligase. (A) Immunoblotting analysis of whole-cell lysates of scrambled siRNA- and synoviolin siRNA-treated LX-2 cells by using anti-synoviolin antibody. The results were derived from 3 independent experiments. (B) The amount of soluble secreted collagen in culture supernatants of scrambled siRNA- and synoviolin siRNA-treated LX-2 cells as quantified by the sircol collagen assay method. The amount of collagen was expressed as a ratio by normalization to the protein concentration in each sample. Data are represented as mean ± SD (n = 3 per group). Unpaired Student's *t*-test was used to evaluate statistical significance. * *P*<0.01. (C) Immunoblotting analysis of whole-cell lysates of transiently transfected LX-2 cells by using anti-KLF6, HA, and β-actin antibodies. The results were derived from 3 independent experiments. (D) Effect of synoviolin on the amount of secreted soluble collagen. The control vector (mock), wt synoviolin-HA, or C307S-HA was co-transfected with the KLF6 vector (KLF6) in LX-2 cells. The amount of secreted soluble collagen in the supernatants of cultured LX-2 cells was quantified by the sircol collagen assay method. The amount of collagen was expressed as a ratio by normalization to the protein concentration in each sample. Data are represented as mean ± SD (n = 3 per group). Unpaired Student's *t*-test was used to evaluate statistical significance. * *P*<0.01, ** *P*<0.001.

To test the hypothesis that synoviolin is directly involved in collagen production in activated HSCs, we analyzed the effect of synoviolin on secreted collagen production using the LX-2 cell line (a human activated HSC cell line) [23]. Because of a major technical limitation in studying primary cultured stellate cells that consistently have very low transfection efficiency—typically less than 1% [23]—we used LX-2 cells that display a relatively high transfection efficiency of >30% using a commercial reagent [23]. To confirm the involvement of synoviolin in collagen production, we initially analyzed the effect of synoviolin siRNA on the amount of secreted collagen using a collagen assay (Fig. 4A). The amount of soluble secreted collagen in the supernatant of synoviolin siRNA-transfected LX-2 cells was ∼30% less than that produced by scrambled siRNA-transfected cells (Fig. 4B).

To observe the functional differences between wt and C307S under conditions of high collagen production, we initially transfected the LX-2 cells with KLF6 cDNA—which is known to induce the expression of type I collagen at the transcriptional level. Then, we transfected the LX-2 cells with empty vector (mock), wt synoviolin (wt), or mutant synoviolin (C307S) lacking its E3 ligase activity, and measured the amount of soluble secreted collagen in the supernatant obtained from the LX-2 cells (Fig. 4C). Wt synoviolin induced a 1.5-fold increase in the amount of secreted collagen, while the mutant synoviolin C307S could not induce any increase in the amount of secreted collagen (Fig. 4D). These results suggest that the enzymatic activity of synoviolin may play a partial role in the regulation of collagen synthesis from activated HSCs.

### Reduced maturation of collagen I proteins in embryonic fibroblasts from Syno^−/−^ mice

**Figure 5:**
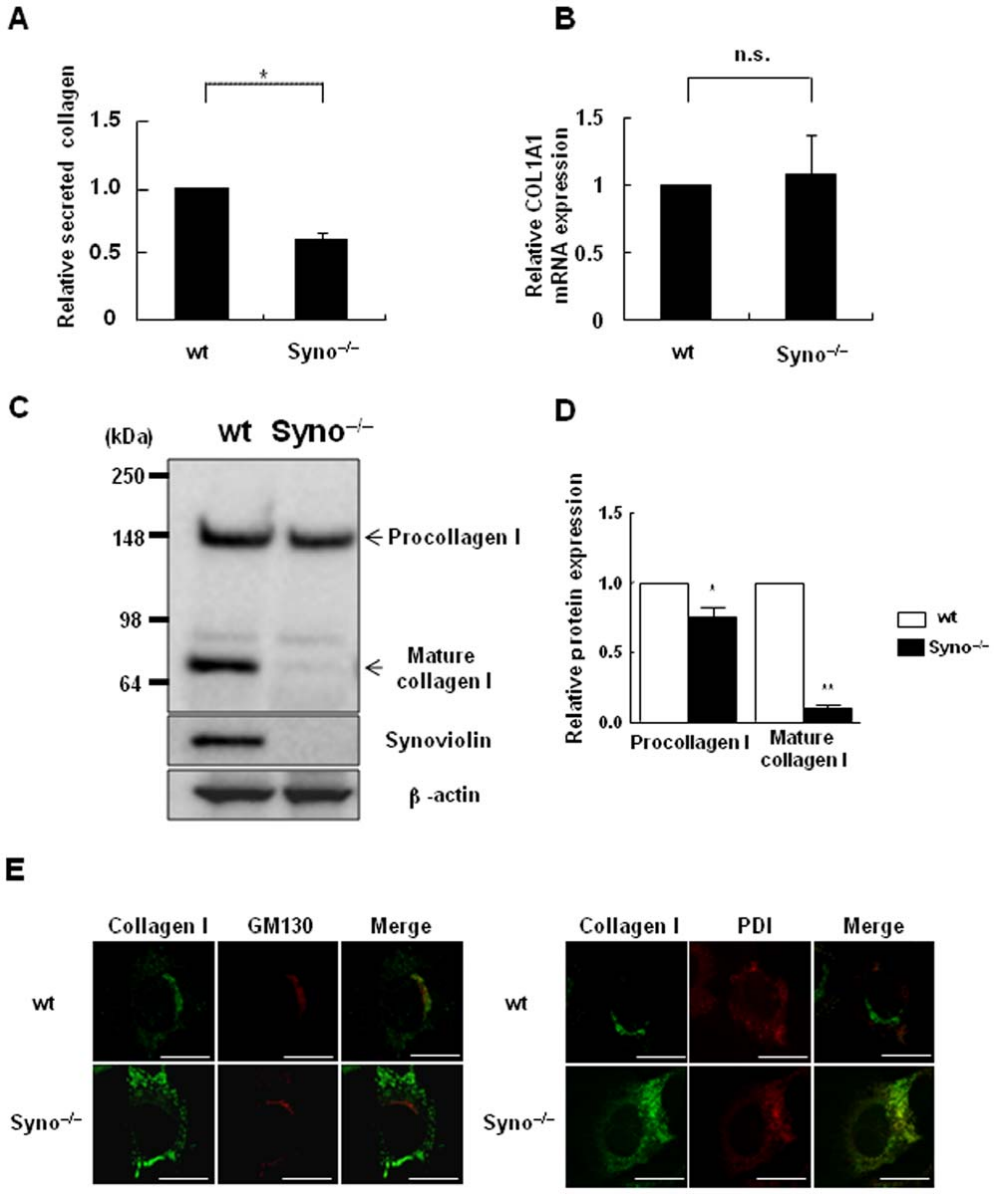
Involvement of synoviolin in collagen synthesis in the maturation of collagen I. (A) Amount of soluble secreted collagen in culture supernatants of wt and Syno^−/−^ MEFs as quantified by the sircol collagen assay method. The amount of collagen was expressed as a ratio by normalization to the protein concentration in each sample. Data are represented as mean ± SD (n = 3 per group). Unpaired Student's *t*-test was used to evaluate statistical significance. * *P*<0.01. Immunoblotting analysis of whole-cell lysates of wt and Syno^−/−^ MEFs by using anti-synoviolin antibody. (B) Effect of synoviolin on COL1A1 mRNA expression in wt and Syno^−/−^ MEFs. The expression level of COL1A1 mRNA was quantified by real-time RT-PCR. The mRNA level was normalized relative to the amount of the transcript of β-actin, a housekeeping gene. Data are represented as mean ± SD (n = 4 per group). Unpaired Student's *t*-test was used for statistical analysis. n.s.  =  not significant. (C) Effect of synoviolin on the expression of intracellular collagen I. The amount of intracellular collagen I in wt and Syno^−/−^ MEFs was analyzed by immunoblot analysis using anti-collagen I, synoviolin, and β-actin antibodies. The results were derived from 5 independent experiments. (D) The amount of procollagen I and mature collagen I in wt and Syno^−/−^ MEFs semiquantified by NIH Image software. Data are represented as mean ± SEM (n = 5 per group). Unpaired Student's *t*-test was used for statistical analysis. * *P*<0.05, ** *P*<0.001. (E) Double immunofluorescence staining was performed using antibodies against type I collagen (AB765P) and GM130 (a marker of *cis*-Golgi compartment) or protein disulfide isomerase (PDI; a marker of ER). The results were derived from 3 independent experiments. Scale bar  = 20 µm.

To investigate the underlying mechanisms, we further analyzed the effect of synoviolin in the process of collagen synthesis using primary Syno^−/−^MEFs (E12.5; Fig. 5). The amount of soluble secreted collagen protein in the supernatants of cultured Syno^−/−^ MEFs was significantly lower than in that of wt MEFs (Fig. 5A), while the mRNA expression levels of the type I collagen gene (*COL1A1*), that is 80% of all types of collagen in HSCs [30], were equivalent between Syno^−/−^ and wt MEFs (Fig. 5B). We further analyzed the amounts of soluble collagen I protein in the whole cell lysates of Syno^−/−^ MEFs using anti-collagen I antibody (Fig. 5C). Interestingly, the amount of cleaved mature collagen I (70 kDa) was significantly decreased, while the amounts of procollagen I (148 kDa) were slightly decreased (Fig. 5C and D). These results suggest that synoviolin is involved in the maturation of collagen I. Since it is known that the procollagen molecules are modified and folded in the ER where synoviolin is located [17] (Fig. S4) and then the mature collagen is transported to the Golgi apparatus [8], we next analyzed the intracellular localization of collagen I in wt and Syno^−/−^ by immunofluorescence staining (Fig. 5E). In wt MEFs, a major portion of the intracellular collagen I was co-localized with GM130, a marker of *cis*-Golgi compartment, while a minor portion was co-localized with PDI—a marker of ER—consistent with previous observations [7], [31]–[33] (Fig. 5E). In contrast, in Syno^−/−^ MEFs, the majority of collagen I was co-localized with PDI, suggesting that the lack of synoviolin caused accumulation of collagen I in the ER. Notably, in Syno^−/−^ MEFs, this abnormal transport was not observed for laminin, which is the one of the proteins secreted via the ER-Golgi apparatus pathway, similar to collagen I (Fig. S5).

## Discussion

In this study, we have demonstrated that the E3 ubiquitin ligase synoviolin is involved in the maturation of collagen I and that reduced synoviolin expression helps attenuate liver fibrogenesis.

The unfolded protein response (UPR), which is a biological defense mechanism of cells, involves translation blocking, chaperone induction, the ERAD system, and the autophagy system [9], [34]–[36]. An insufficient UPR results in the accumulation of unfolded proteins in the ER, which may induce ER stress-mediated apoptosis. ERAD is an ATP-dependent ubiquitin-proteasome process that functions to reduce the burden of excess unfolded proteins in the ER [9]. Thus, ERAD is important for the maintenance of homeostasis in living organisms. Several E3 ubiquitin ligases have already been identified, and their vital functions have been intensively studied in recent years. Current research indicates the association of the loss of function of E3 ubiquitin ligases with ERAD and associated disorders. For example, a loss-of-function mutation of the *parkin* gene, which encodes a well-known E3 ubiquitin ligase, results in neuronal death in the substantia nigra of patients with autosomal recessive juvenile Parkinsonism [37]. On the other hand, we have previously demonstrated the association of the hyperfunctioning of E3 ubiquitin ligases with ERAD and the associated disorders; the overexpression of synoviolin, an E3 ubiquitin ligase that functions in the ERAD system, is a pathogenic factor in arthropathy [18]. Synoviolin is also strongly expressed in rheumatoid synovial cells and is involved in the pathogenesis of rheumatoid arthritis (RA) [11]–[14]. These data suggest that synoviolin overexpression leads to ERAD hyperactivation in rheumatoid synovial cells, implicating the hyperfunctioning of the ERAD system as an important pathogenic mechanism in diseases, which is a novel concept [14]. In this study, we noted that synoviolin overexpression led to excess secretion of mature collagen from activated HSCs, while reduced synoviolin expression resulted in resistance to liver fibrogenesis. This initial report suggests that hyperactivity of the ERAD system due to the overexpression of an E3 ubiquitin ligase may cause liver fibrosis. Our results also support the idea of the hyperfunctioning of the ERAD system as an important pathogenic mechanism in liver diseases. Further, our results also suggest that modifications in ERAD activity may control the fibrogenic potential of HSCs following liver injury.

In our previous study, we reported that mouse embryonic fibroblasts from Syno^−/−^ are selectively susceptible to ER stress-induced apoptosis. The MEFs derived from the Syno^−/−^ embryos exhibited high and selective susceptibility to ER stress *in vitro* along with increased expressions of ER stress-inducible proteins, including Bip/Grp78 and CHOP/Gadd153 [22]. These data suggest that ER stress occurs in Syno^−/−^ MEFs. In this study, the number of apoptotic activated HSCs was increased in Syno^+/−^ mice as compared to those in wt mice in liver fibrosis models using CCl_4_, which is a well-known ER stress inducer (Fig. 3D). Under such severe ER stress conditions, ER stress may lead to programmed cell death in HSCs; subsequently, the accumulation of extracellular matrix would be attenuated.

Collagen is essential for tissue repair and regeneration in liver injury. However, when liver damage persists because of chronic injury, collagen is produced and secreted in excess by activated HSCs [38]. Further, among various collagen proteins, collagen I comprises approximately 90% of the body's total collagen [39]. However, it is not well understood how collagen is translated, modified after translation, processed, correctly folded, and secreted as collagen triple helix. In HSP47-deficient cells, collagen triple helix formation and stability are impaired, and the improperly folded triple helices form insoluble aggregates in the ER [7]. In addition, the role of ERAD in the degradation of mutant unfolded procollagen I caused by a mutation in the collagen I gene has also been recently discovered [34]–[36], [40]. Further, the polyubiquitination of procollagen I (148 kDa) was detected in Mov-IAFS cell lines, and the mutation was found to be located in the C-propeptide region of the procollagen I chain using an *in vivo* ubiquitination assay [35]. These reports suggest that the posttranslational regulation of collagen expression is crucial in collagen synthesis. In this study, we found that the amount of cleaved mature collagen I (70 kDa) was significantly decreased, while the amount of procollagen I (148 kDa) was not increased by the lack of synoviolin (Fig. 5D). Furthermore, lack of synoviolin also caused an accumulation of collagen I in the ER (Fig. 5E). These data suggest that synoviolin can be involved in the maturation process of procollagen I synthesis between the ER and *cis*-Golgi compartment. ER-resident E3 ubiquitin ligases, including synoviolin, are crucial in the ERAD system because E3 ubiquitin ligase directly binds to specific target substrates and adds a polyubiquitin chain. Therefore, we hypothesize that synoviolin may regulate the quality of collagen protein through the polyubiquitination of the unfolded procollagen directly or may regulate the quality of these collagen-specific molecular chaperones indirectly via its ubiquitination and degradation pathways. We are currently investigating the collagen-specific target substrates of synoviolin.

In conclusion, we have demonstrated that the E3 ubiquitin ligase synoviolin is essential to the maturation of collagen I. Using experiments involving a liver fibrosis model with Syno^+/−^ mice, we have also demonstrated that synoviolin is involved in liver fibrogenesis via apoptosis and collagen synthesis of the activated HSCs. These data emphasize the novel role of the E3 ubiquitin ligase synoviolin in liver fibrosis.

## Supporting Information

Figure S1 Double-labeled fluorescent immunohistochemical analysis of rat mammary tissue sections as positive and negative controls for TUNEL staining. The expression and localization of TUNEL (green) and α-SMA (red) were analyzed using an in situ apoptosis assay kit and α-SMA antibodies in positive (right panels) and negative controls (left panels) using rat mammary tissue sections. The nuclei were counterstained with DAPI (Vectashield). Scale bar  = 200 µm.
(2.23 MB TIF)

Figure S2 HE staining of liver tissue and serum biochemical tests for wt and Syno+/− mice in natural conditions. (A) HE staining of mouse liver sections from wt and Syno+/− mice in natural conditions. Scale bar  = 200 µm. (B) The serum levels of aspartate aminotransferase (AST), alanine aminotransferase (ALT), lactate dehydrogenase (LDH), total bilirubin (T-bil), total protein, albumin (Alb), and the serum albumin/globulin (A/G) ratio for wt (n = 6) and Syno+/− mice (n = 7) in the natural condition. Data are represented as mean ± SEM. Serum biochemical tests were performed using an outsourced examination (Mitsubishi Chemical Medience Corporation, Tokyo, Japan). We measured aspartate aminotransferase (AST), alanine aminotransferase (ALT), lactate dehydrogenase (LDH), the total bilirubin (T-bil), total protein (TP), albumin (Alb), and the albumin/globulin (A/G) ratio using mice serum. The liver sections were deparaffinized, stained with hematoxylin and eosin (H&E), and evaluated using light microscopy. We performed the serum biochemical tests within 48 h after completion of the chronic CCl4 protocol.
(1.44 MB TIF)

Figure S3 Serum biochemical tests for wt and Syno+/− mice in the CCl4-induced chronic hepatic injury model. The serum levels of aspartate aminotransferase (AST), alanine aminotransferase (ALT), lactate dehydrogenase (LDH), total bilirubin (T-bil), total protein, albumin (Alb), and the serum albumin/globulin (A/G) ratio for wt (n = 14) and Syno+/− mice (n = 12) of the CCl4-induced chronic hepatic injury model. Data are represented as mean ± SEM.
(0.47 MB TIF)

Figure S4 Double immunofluorescence staining was performed in wt and Syno−/− MEFs using antibodies against synoviolin and PDI. Fluorescent images were visualized using a Zeiss LSM 510 META confocal fluorescence microscope (Carl Zeiss, Jena, Germany). The results were derived from 3 independent experiments. Scale bar  = 20 µm.
(0.78 MB TIF)

Figure S5 The expression and localization of the laminin protein in wt and Syno−/− MEFs. (A) Effect of synoviolin on the expression of laminin. The amount of laminin in whole cell lysates of wt and Syno−/− MEFs was analyzed by immunoblot analysis using anti-laminin, synoviolin, and β-actin antibodies. The antibodies used in this study were as follows: anti-laminin polyclonal antibody (Sigma). The results were derived from 3 independent experiments. (B) Double immunofluorescence staining was performed using antibodies against laminin and GM130 or PDI. Fluorescent images were visualized using a Zeiss LSM 510 META confocal fluorescence microscope (Carl Zeiss, Jena, Germany). The results were derived from 3 independent experiments. Scale bar  = 20 µm.
(0.96 MB TIF)
