# Supplementary figures and images for: E3 Ubiquitin Ligase Synoviolin Is Involved in Liver Fibrogenesis
Source: PLoS One. 2010 Oct 25;5(10):e13590. doi: 10.1371/journal.pone.0013590 (PMC2963597; doi:10.1371/journal.pone.0013590)

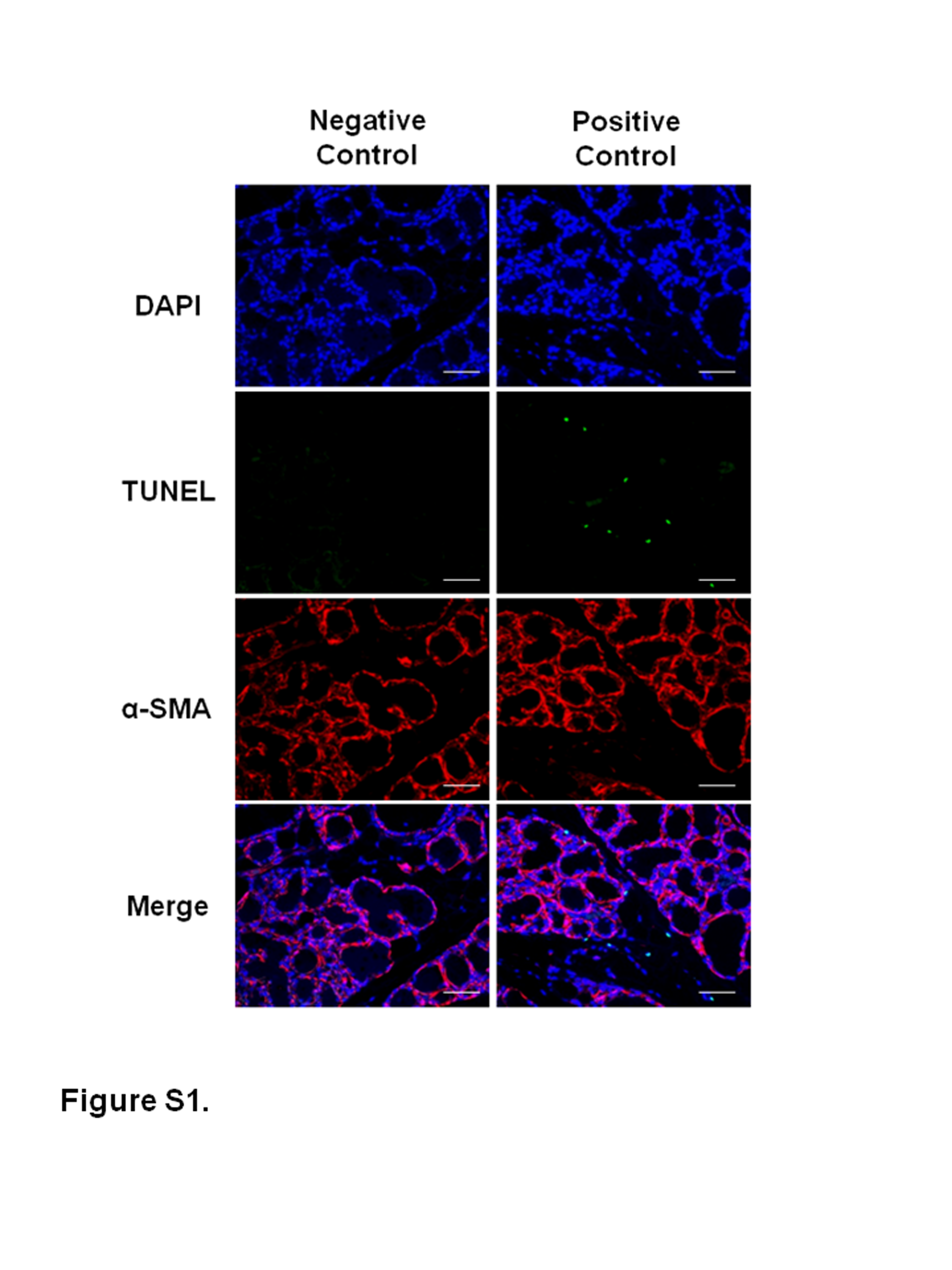

Supplement: Figure S1 — Double-labeled fluorescent immunohistochemical analysis of rat mammary tissue sections as positive and negative controls for TUNEL staining. The expression and localization of TUNEL (green) and α-SMA (red) were analyzed using an in situ apoptosis assay kit and α-SMA antibodies in positive (right panels) and negative controls (left panels) using rat mammary tissue sections. The nuclei were counterstained with DAPI (Vectashield). Scale bar = 200 µm. (2.23 MB TIF) [file pone.0013590.s001.tif]

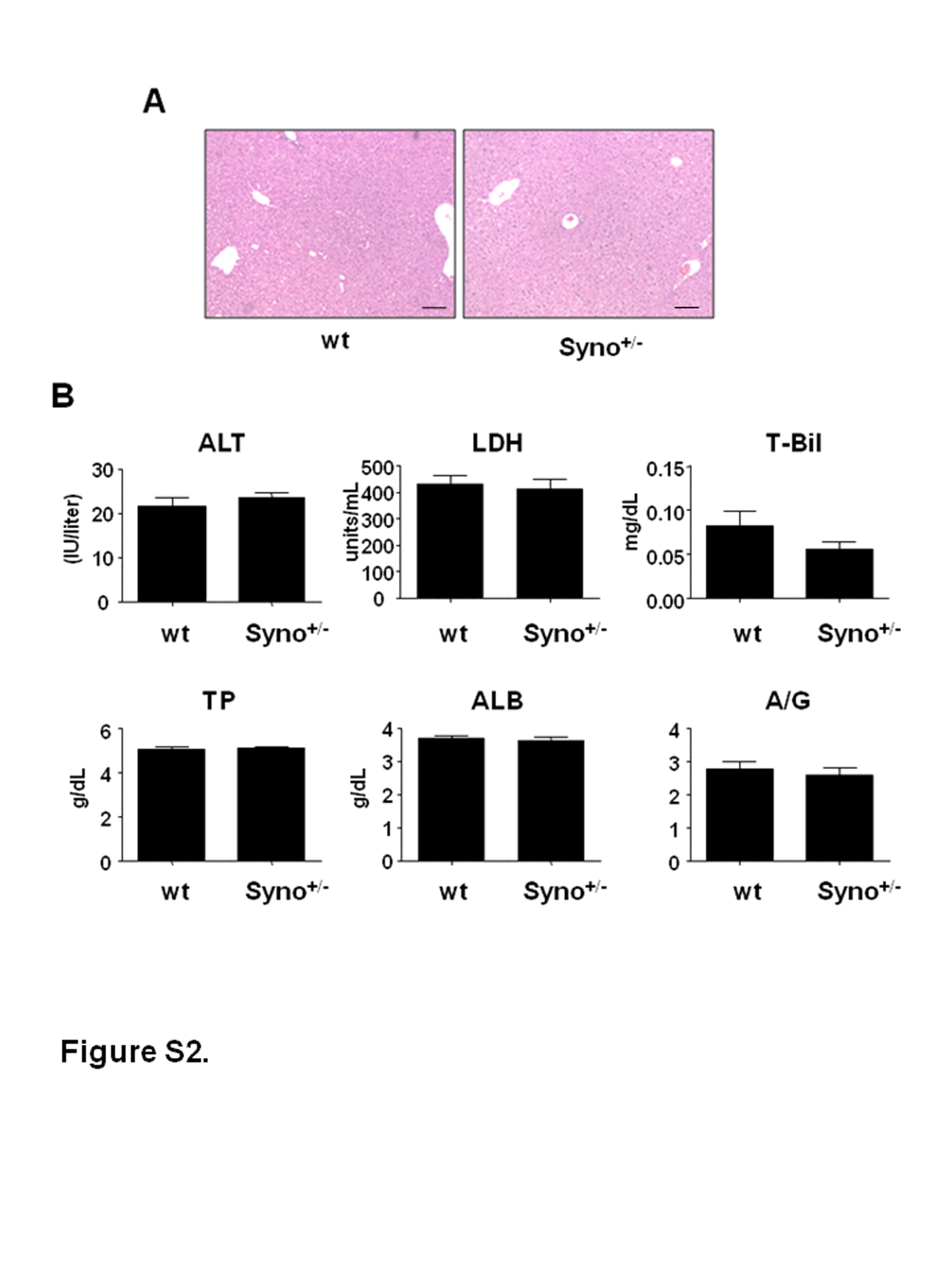

Supplement: Figure S2 — HE staining of liver tissue and serum biochemical tests for wt and Syno+/− mice in natural conditions. (A) HE staining of mouse liver sections from wt and Syno+/− mice in natural conditions. Scale bar = 200 µm. (B) The serum levels of aspartate aminotransferase (AST), alanine aminotransferase (ALT), lactate dehydrogenase (LDH), total bilirubin (T-bil), total protein, albumin (Alb), and the serum albumin/globulin (A/G) ratio for wt (n = 6) and Syno+/− mice (n = 7) in the natural condition. Data are represented as mean ± SEM. Serum biochemical tests were performed using an outsourced examination (Mitsubishi Chemical Medience Corporation, Tokyo, Japan). We measured aspartate aminotransferase (AST), alanine aminotransferase (ALT), lactate dehydrogenase (LDH), the total bilirubin (T-bil), total protein (TP), albumin (Alb), and the albumin/globulin (A/G) ratio using mice serum. The liver sections were deparaffinized, stained with hematoxylin and eosin (H&E), and evaluated using light microscopy. We performed the serum biochemical tests within 48 h after completion of the chronic CCl4 protocol. (1.44 MB TIF) [file pone.0013590.s002.tif]

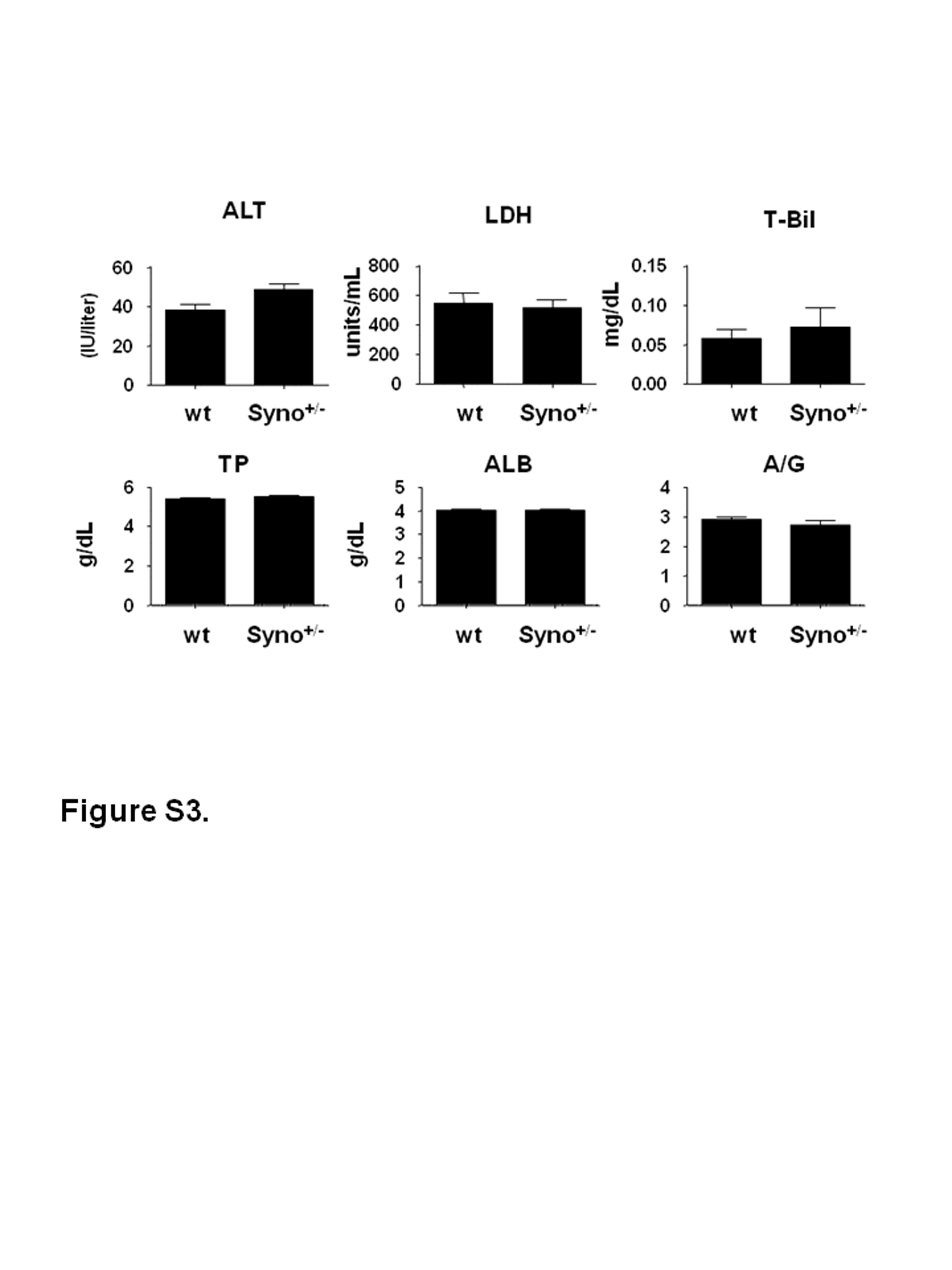

Supplement: Figure S3 — Serum biochemical tests for wt and Syno+/− mice in the CCl4-induced chronic hepatic injury model. The serum levels of aspartate aminotransferase (AST), alanine aminotransferase (ALT), lactate dehydrogenase (LDH), total bilirubin (T-bil), total protein, albumin (Alb), and the serum albumin/globulin (A/G) ratio for wt (n = 14) and Syno+/− mice (n = 12) of the CCl4-induced chronic hepatic injury model. Data are represented as mean ± SEM. (0.47 MB TIF) [file pone.0013590.s003.tif]

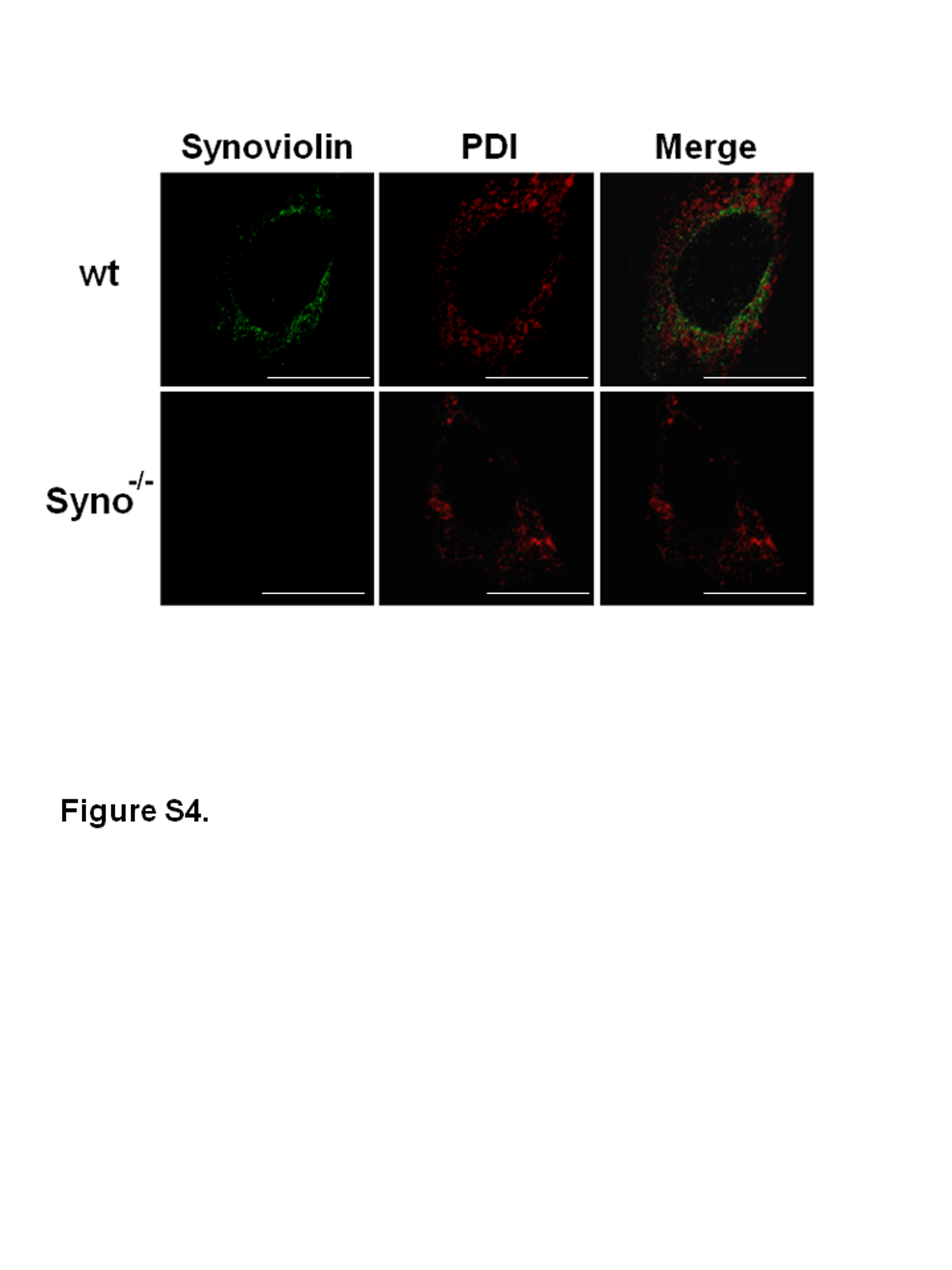

Supplement: Figure S4 — Double immunofluorescence staining was performed in wt and Syno−/− MEFs using antibodies against synoviolin and PDI. Fluorescent images were visualized using a Zeiss LSM 510 META confocal fluorescence microscope (Carl Zeiss, Jena, Germany). The results were derived from 3 independent experiments. Scale bar = 20 µm. (0.78 MB TIF) [file pone.0013590.s004.tif]

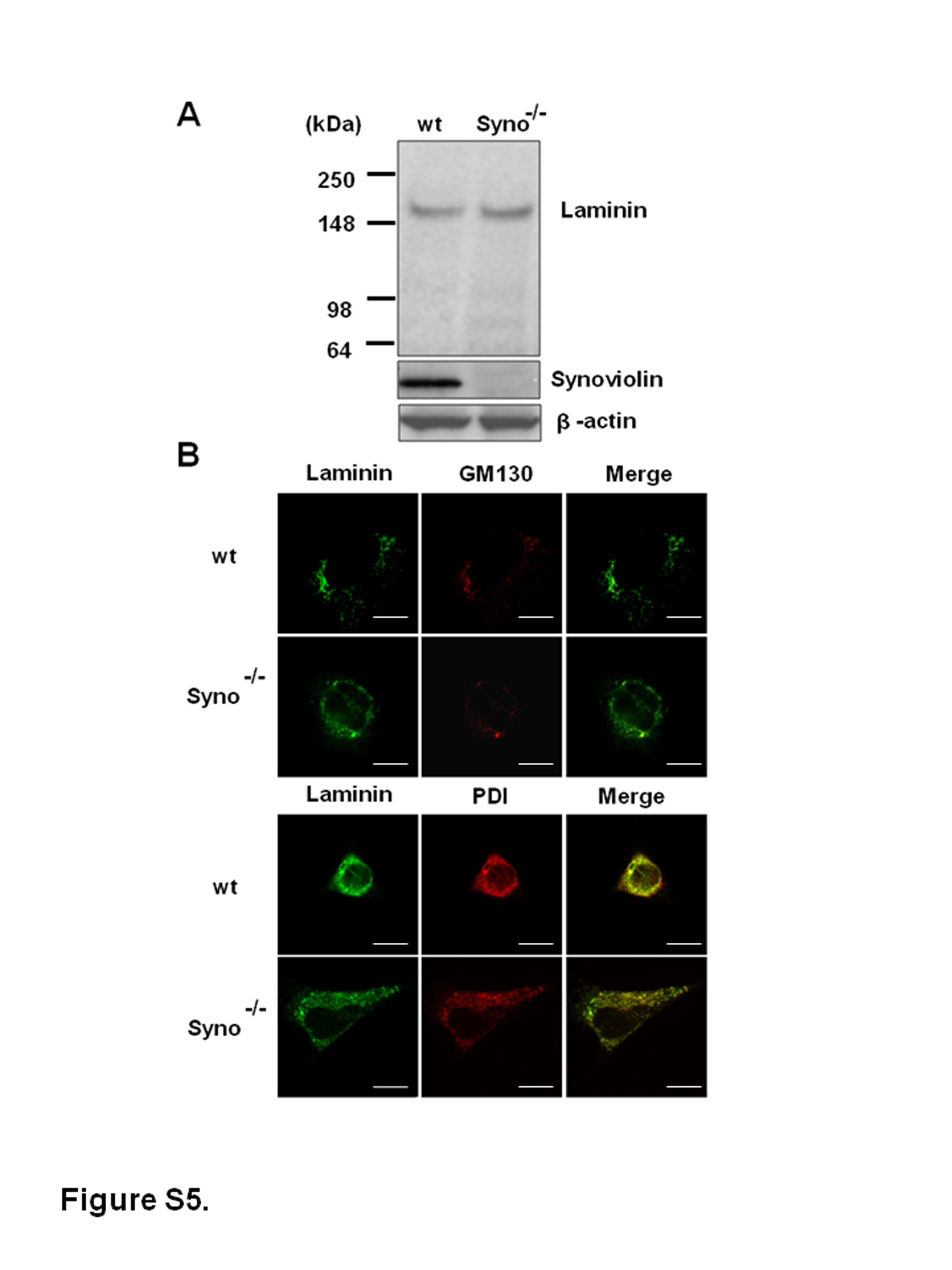

Supplement: Figure S5 — The expression and localization of the laminin protein in wt and Syno−/− MEFs. (A) Effect of synoviolin on the expression of laminin. The amount of laminin in whole cell lysates of wt and Syno−/− MEFs was analyzed by immunoblot analysis using anti-laminin, synoviolin, and β-actin antibodies. The antibodies used in this study were as follows: anti-laminin polyclonal antibody (Sigma). The results were derived from 3 independent experiments. (B) Double immunofluorescence staining was performed using antibodies against laminin and GM130 or PDI. Fluorescent images were visualized using a Zeiss LSM 510 META confocal fluorescence microscope (Carl Zeiss, Jena, Germany). The results were derived from 3 independent experiments. Scale bar = 20 µm. (0.96 MB TIF) [file pone.0013590.s005.tif]
